# Abatacept in rheumatoid arthritis: survival on drug, clinical outcomes, and their predictors—data from a large national quality register

**DOI:** 10.1186/s13075-020-2100-y

**Published:** 2020-01-22

**Authors:** Giovanni Cagnotto, Minna Willim, Jan-Åke Nilsson, Michele Compagno, Lennart T. H. Jacobsson, Saedis Saevarsdottir, Carl Turesson

**Affiliations:** 1 Rheumatology, Department of Clinical Sciences, Lund University, Malmö, Sweden; 2 Department of Rheumatology, Skåne University Hospital, Lund, Sweden; 3 Rheumatology, Department of Clinical Sciences, Lund University, Lund, Sweden; 4 Department of Rheumatology & Inflammation Research, The Sahlgrenska Academy, University of Gothenburg, Gothenburg, Sweden; 5 Unit of Translational Epidemiology, Institute of Environmental Medicine, Karolinska Institutet, Stockholm, Sweden; 6 Rheumatology Unit, Department of Medicine, Karolinska Institutet, Solna, Stockholm, Sweden; 7 Department of Rheumatology, Skåne University Hospital, Malmö, Sweden

**Keywords:** Rheumatoid arthritis, Abatacept, Survival on drug, Treatment outcome, Response predictors

## Abstract

**Background:**

There are limited data regarding efficacy of abatacept treatment for rheumatoid arthritis (RA) outside clinical trials. Quality registers have been useful for observational studies on tumor necrosis factor inhibition in clinical practice. The aim of this study was to investigate clinical efficacy and tolerability of abatacept in RA, using a national register.

**Methods:**

RA patients that started abatacept between 2006 and 2017 and were included in the Swedish Rheumatology Quality register (*N* = 2716) were investigated. Survival on drug was estimated using Kaplan-Meier analysis. The European League Against Rheumatism (EULAR) good response and Health Assessment Questionnaire (HAQ) response (improvement of ≥ 0.3) rates (LUNDEX corrected for drug survival) at 6 and at 12 months were assessed. Predictors of discontinuation were investigated by Cox regression analyses, and predictors of clinical response by logistic regression. Significance-based backward stepwise selection of variables was used for the final multivariate models.

**Results:**

There was a significant difference in drug survival by previous biologic disease-modifying antirheumatic drug (bDMARD) exposure (*p* < 0.001), with longer survival in bionaïve patients. Men (hazard ratio (HR) 0.86, 95% confidence interval (CI) 0.74–0.98) and methotrexate users (HR 0.85, 95% CI 0.76–0.95) were less likely to discontinue abatacept, whereas a high pain score predicted discontinuation (HR 1.14 per standard deviation, 95% CI 1.07–1.20). The absence of previous bDMARD exposure, male sex, and a low HAQ score were independently associated with LUNDEX-corrected EULAR good response. The absence of previous bDMARD exposure also predicted LUNDEX-corrected HAQ response.

**Conclusions:**

In this population-based study of RA, bDMARD naïve patients and male patients were more likely to remain on abatacept with a major clinical response.

## Background

Rheumatoid arthritis (RA) is a chronic autoimmune disease that may lead to progressive joint damage and disability. In the last decade, outcomes of RA have improved considerably due to the recognition of the benefits of an early and aggressive treatment with disease-modifying antirheumatic drugs (DMARDs), with a structured follow-up, and to the development of biologic DMARD (bDMARDs). Disease remission or at least reduction in joint inflammation, prevention of joint damage, and preservation of function is now possible [1–4].

The rheumatology community, industry, and regulators have recognized the need for observational studies to monitor the safety of biologic therapy. Academia-initiated biologic registers are key not only for pharmacovigilance, but also to examine long-term effectiveness. Efficacy results observed in placebo-controlled randomized trial are sometimes different from the real-world effectiveness data. This can be due to patient selection, adherence to therapy, or other factors (i.e., Hawthorne effect) [5, 6].

Abatacept is a bDMARD acting through a selective inhibition of T cell co-stimulation. It was approved for RA treatment in Europe in 2007 (but available in Sweden for use in some patients already in 2006). Initially, it was listed in recommendations as an option only in patients with inadequate response to tumor necrosis factor (TNF) inhibitors [7], but it has subsequently been recommended as one of the first line bDMARDs for RA [8].

The Swedish Rheumatology Quality (SRQ) register has been used to study the effectiveness of TNF inhibitor switch in RA patient on a national level in Sweden [9]. It has also been included in previous international collaborations between European registers for studies of treatment with rituximab [10] and abatacept [11] for RA. Data on patients treated with abatacept (characteristics, diagnosis, previous treatment, and outcomes) have been collected in the SRQ register since abatacept was first available in 2006.

There is limited data on abatacept efficacy and on predictors of clinical response in real-world as derived from a single national register. Most studies are based on pooled data from several registers [12] or multicenter studies from several countries [13]. Such studies may be affected by patient heterogeneity and differences in access to bDMARDs [12].

The aim of this study was to describe survival on drug and clinical effectiveness in RA patients treated with abatacept and to investigate predictors of remaining on treatment and having a significant clinical response, in a national cohort.

## Patients and methods

### Study design

This was an observational study, based on a nationwide clinical RA register [14]. Clinical effectiveness was assessed as proportions of patients remaining on abatacept over time (drug survival) and as the proportions of patients remaining on therapy and achieving predefined standard clinical outcomes—according to the LUNDEX method [15], which has been used in several register-based studies of bDMARD effectiveness [16, 17].

### Source population

The Swedish Rheumatology Quality register (SRQ) is a nationwide clinical register in Sweden of patients with chronic inflammatory joint diseases, including RA. RA patients starting bDMARDs have been included in the SRQ since 1999. The coverage of SRQ has been estimated at 87% of bDMARD treated patients, with no indications of compromised external generalizability regarding demography [18].

The SRQ covers clinical information on disease characteristics and antirheumatic treatment, prospectively recorded at treatment initiation and at subsequent visits. Dates of starting and stopping treatment, and the cause of discontinuing treatment are recorded by the physician who manages the patient at each visit, as part of regular clinical care. Studies on bDMARDs in the SRQ are coordinated by the Anti-Rheumatic Therapy in Sweden (ARTIS) Study group.

Patients with diagnosis of RA registered in the SRQ and starting abatacept treatment between April 2006 and November 2017 were included in the study. Clinical data from the SRQ were collected through data capture for the period 1 April 2006 to 30 November 2017.

### Survival on drug

Survival on drug was estimated as the time to registered discontinuation of abatacept. Median time to discontinuation and estimated proportions still treated with abatacept at 6 and 12 months after treatment initiation were derived. Patients lost to follow-up (died, migrated from Sweden, or excluded from the SRQ for other reasons) or still treated with abatacept at the time of data capture were right censored. Reasons for abatacept discontinuation were also collected.

### Clinical response

Clinical effectiveness of abatacept was evaluated by means of the European League Against Rheumatism (EULAR) response [19, 20] and by Health Assessment Questionnaire (HAQ) disability index (HAQ-DI) response [21], defined as improvement in HAQ score ≥ 0.3 [22]. Baseline was defined as time of abatacept treatment start. In addition, proportions achieving disease activity score in 28 joints (DAS28) defined remission (DAS28 < 2.6) or low disease activity (DAS28 ≤ 3.2) [20] were calculated. Efficacy outcomes were evaluated at 6 months (i.e., the visit closest to 180 days, and within 150 to 240 days, after baseline visit) and at 12 months (i.e., the visit closest to 365 days, and within 300 to 450 days, after baseline visit). Moreover, patients achieving LUNDEX-corrected clinical outcomes were defined as those remaining on the drug and achieving the outcome [15]. Patients who discontinued abatacept treatment before the follow-up time points 6 and 12 months were considered non-responders for the corresponding LUNDEX-corrected outcome measures. In addition, LUNDEX-corrected proportions of clinical responders were also calculated as the fraction of patients remaining on treatment (including those with missing clinical data) multiplied by the proportion achieving the outcome among those with data available [15].

### Exposures

Candidate predictors of drug survival and clinical effectiveness registered at baseline were age, sex, patient-reported pain on a visual analog scale (VAS), DAS28 and DAS28-CRP, HAQ-DI, disease duration, route of abatacept administration, concomitant treatment with conventional synthetic DMARDs (csDMARDs), with methotrexate (MTX), with glucocorticoids, and previous bDMARD exposure. Three categories of patients were defined based on information collected in the SRQ prior to abatacept initiation: bionaïve patients and those with 1 previous bDMARD or ≥ 2 previous bDMARDs.

### Statistics

Survival on drug up to 5 years, by previous exposure to bDMARDs, was estimated using the Kaplan-Meier method (log-rank test). Predictors for drug discontinuation were investigated in Cox proportional hazards analyses, and for LUNDEX-corrected EULAR and HAQ responses in logistic regression models. In analyses of the relation between previous bDMARD exposure and outcomes, those who had been treated with ≥ 2 bDMARDs (the largest category) were used as the reference. Variables with a *p* value < 0.20 in these analyses were retained for the starting multivariate model. Previous exposure to bDMARD treatment was forced in the model as an exposure variable. Significance-based backward stepwise selection of variables was used for the final multivariate model. In sensitivity analyses, all covariates with a *p* value of < 0.10 in the univariate models were included in the multivariate models. Furthermore, we also performed analyses including all covariates. Models were constructed with and without exclusion of covariates due to collinearity (*r* > 0.3, Pearson’s test or Spearman’s test, as appropriate).

## Results

### Patient characteristics

A total of 2716 patients with RA starting abatacept during the study period were included. Seventeen percent were bionaïve, 27% had been exposed to 1 previous bDMARD, and 56% to ≥ 2 previous bDMARDs. About half of the patients had intravenous administration of abatacept when first starting treatment. The mean disease duration at treatment start was 14.2 years. Most patients had active disease, with mean values for DAS28-CRP and HAQ-DI of 4.66 and 1.25, respectively. Variables reflecting disease activity and disease severity were comparable between the three categories of bDMARD exposure (Table 1). However, there were some differences in sex (*p* < 0.001), disease duration (*p* < 0.001), route of abatacept administration (*p* < 0.002), and glucocorticoids treatment (*p* < 0.001). Seventy-two per cent of patients in the bDMARD naïve group were women, while about 80% were women in the bDMARDs experienced groups. Bionaïve patients had a shorter disease duration (mean 9.5 years) in comparison with patients exposed to 1 previous bDMARD (mean 14.4 years) and to ≥ 2 previous bDMARDs (mean 15.5 years). Forty-three percent of bionaïve patients were treated with intravenous abatacept compared with 52% of the bDMARD experienced patients. Less bionaïve patients were treated with glucocorticoids (39%) in comparison with bDMARD experienced patients (47% and 52% in the 2 groups, respectively). The complete baseline characteristics of the cohort are shown in Table 1.

**Table 1:** Clinical characteristics at baseline visit by number of previous bDMARDs

	Total	Bionaïve	1 previous bDMARD	≥ 2 previous bDMARDs
Number of patients (%)	2716	453 (16.7)	741 (27.3)	1522 (56)
Female sex (%)	2176 (80.1)	325 (71.7)	599 (80.8)	1252 (82.3)
Age at treatment start (years); mean (SD)	59.3 (13.3)	61.7 (14.0)	60.7 (12.9)	57.8 (13.0)
Duration of RA at treatment start (years); mean (SD)	14.2 (11.4)	9.5 (11.1)	14.4 (11.8)	15.5 (10.8)
Intravenous treatment	1365 (50.3%)	194 (42.8%)	381 (51.8%)	790 (52.1%)
Subcutaneous treatment	1338 (49.3%)	257 (57.0%)	355 (48.2%)	726 (47.9%)
ESR (mm 1st h); median (IQR)	23 (11–42)	23 (12–42)	23.5 (12–40.25)	22 (10–41)
CRP (mg/l); median (IQR)	9 (3.5–23)	11 (5–24)	8 (3.48–23)	8 (3–22)
DAS28; mean (SD)	4.98 (1.29)	5.01 (1.23)	4.93 (1.28)	4.99 (1.31)
DAS28-CRP; mean (SD)	4.66 (1.13)	4.64 (1.14)	4.57 (1.13)	4.70 (1.13)
VAS pain (0–100); mean (SD)	60 (23)	58 (24)	59 (23)	62 (22)
VAS global (0–100); mean (SD)	60 (22)	56 (23)	60 (23)	62 (22)
Swollen joint count (0–28); median (IQR)	5 (2–9)	6 (3–10)	5 (2–8)	5 (2–9)
Tender joint count (0–28); median (IQR)	6 (3–10)	6 (2–11)	6 (3–10)	6 (3–11)
HAQ-DI (0–3); mean (SD)	1.32 (0.63)	1.16 (0.63)	1.30 (0.65)	1.37 (0.62)
Physicians global (0–4); median (IQR)	2 (2–3)	2 (2–3)	2 (2–3)	2 (2–3)
Current methotrexate	1288 (57%)	196 (55%)	373 (61%)	719 (55%)
Current glucocorticoids	1316 (49%)	176 (39%)	345 (47%)	795 (52%)
Glucocorticoids dose in mg, prednisolone equivalent; mean (SD)	7.5 (4.2)	7.6 (3.9)	6.9 (4.0)	7.8 (4.3)
Current csDMARD	1489 (55%)	237 (52%)	425 (57%)	827 (54%)

Missing data: Duration of RA at treatment start (years), 17; intravenous/subcutaneous treatment, 13; ESR, 714; CRP, 580; DAS 28, 921; CRP, 811; VAS pain, 748; VAS global, 711; swollen joint count, 624; tender joint count, 625; HAQ-DI, 825; physician global, 711; current methotrexate, 439

### Survival on drug

**Fig. 1:**
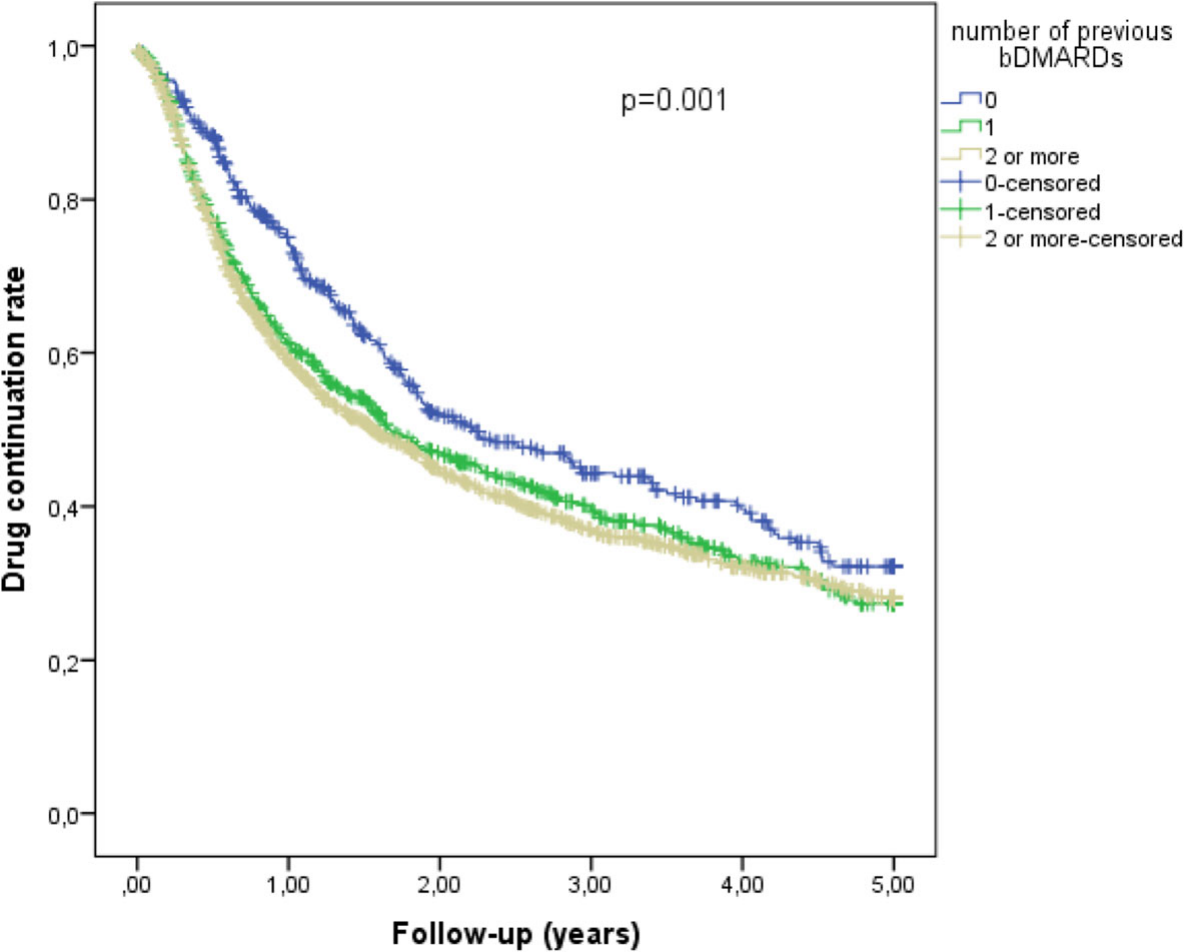
Survival on abatacept by previous bDMARD exposure. Drug continuation rates in patients treated with no previous bDMARD, 1 previous bDMARD, and ≥ 2 previous bDMARDs. Significant difference (*p* = 0.001, log-rank test) due to lower abatacept discontinuation in patients with no previous bDMARDs compared to those with 1 or ≥ 2 previous bDMARDs

Overall, 75% of the patients remained on treatment with abatacept at 6 months, and 55% at 12 months. The corresponding proportions were 85% and 64% for bionaïve patients, 74% and 54% for those with 1 previous bDMARD exposure, and 73% and 52% for those exposed to ≥ 2 previous bDMARDs. Overall, 50.0% of discontinuations were due to insufficient drug effect, 18.1% to side effects, 2.5% to persistent disease remission, and 29.4% to other reasons (non-specified reason, patient preference, pregnancy, death, etc). Median survival on abatacept was 1.74 years (95% confidence interval (CI) 1.58–1.90), 2.23 years for bionaïve patients (95% CI 1.69–2.76), 1.68 years for those exposed to 1 previous bDMARD (95% CI 1.34–2.01), and 1.56 years for those exposed to ≥ 2 previous bDMARDs (95% CI 1.35–1.76). There was a statistically significant difference in survival on drug between bionaïve and bDMARD experienced patients (*p* = 0.001, Fig. 1).

Bionaïve patients were less likely to discontinue treatment over time compared to those who had been treated with ≥ 2 bDMARDs, whereas there was no difference between the subsets of bDMARD experienced patients (Table 2). In univariate analyses, male sex, lack of previous exposure to bDMARDs, and baseline treatment with methotrexate predicted longer survival on abatacept (Table 2). Moreover, higher DAS28-CRP, higher VAS pain, and higher HAQ score at baseline-predicted abatacept discontinuation (Table 2). In the multivariate model with significance-based backward stepwise selection of variables, male sex (hazard ratio (HR) 0.86; 95% CI 0.74–0.98), VAS pain (HR 1.14 per standard deviation (SD); 95% CI 1.07–1.20), and baseline treatment with MTX (HR 0.85; 95% CI 0.76–0.95) had significant independent effects on abatacept discontinuation (Table 2).

**Table 2:** Predictors of abatacept discontinuation. Cox regression analysis

	Unadjusted analysis HR (95% CI)	Multivariate analysis—final model HR (95% CI)
Sex		
Male	0.85 (0.75–0.96)	0.86 (0.74–0.98)
No of previous bDMARDs		
≥ 2 bDMARDs	Reference (1.0)	*
Bionaïve	0.78 (0.68–0.90)	*
1 bDMARD	0.94 (0.84–1.05)	*
Baseline clinical characteristics		
DAS28-CRP (per SD)	1.11 (1.04–1.17)	*
VAS pain (per SD)	1.14 (1.08–1.21)	1.14 (1.07–1.20)
Current Methotrexate	0.86 (0.78–0.96)	0.85 (0.76–0.95)
HAQ-DI (per SD)	1.10 (1.04–1.17)	*
Age (per SD)	0.99 (0.94–1.04)	*
Disease duration (per SD)	0.98 (0.93–1.03)	*
Current glucocorticoids	1.08 (0.98–1.19)	*
Current csDMARD	0.93 (0.85–1.03)	*
i.v. abatacept administration	1.02 (0.92–1.12)	*

*Not included in the final model. The first multivariate model in the stepwise analysis included sex, bDMARD exposure, DAS28-CRP, VAS pain, methotrexate at baseline, HAQ-DI, glucocorticoids at baseline. Multivariate model includes 1768 patients

Results were similar in sensitivity analyses including all covariates or all covariates with *p* < 0.10 in the univariate models, with and without exclusion of covariates due to collinearity, except that the association between MTX and discontinuation did not reach statistical significance in the model that did not exclude covariates based on multicollinearity (see Additional file 1: Table S1 and S2).

### Clinical response

**Fig. 2:**
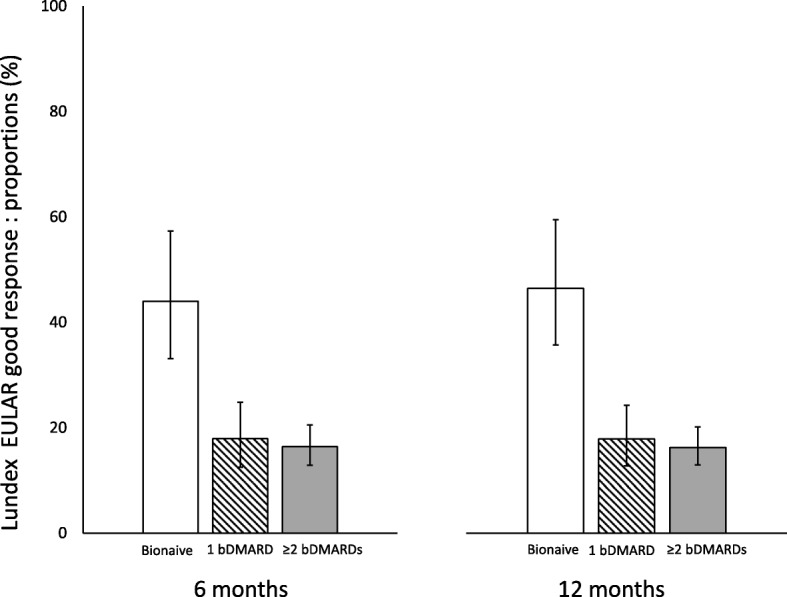
Proportion of patients achieving LUNDEX-corrected EULAR good response by previous bDMARD exposure. *p* < 0.001 for bionaïve patients vs patients treated with 1 and with ≥ 2 previous bDMARDs. Bars are 95% CI

**Fig. 3:**
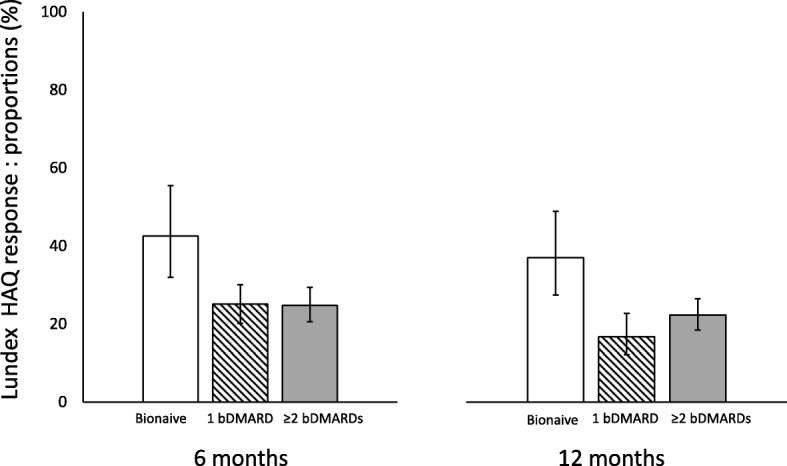
Proportion of patients achieving LUNDEX-corrected HAQ response by previous bDMARD exposure. *p* < 0.002 for bionaïve patients vs patients treated with 1 and with ≥ 2 previous bDMARDs. Bars are 95% CI

Twenty-four percent of the patients achieved a EULAR good response at 6 months and 29% at 12 months (see Additional file 1: Table S3). The corresponding proportions with a EULAR good or moderate response were 59% and 62%, and with a HAQ response were 31% and 33%, respectively (see Additional file 1: Table S3 and S4). Among all patients initiating abatacept, 21% were still on treatment and achieved a EULAR good response at 6 months and at 12 months (LUNDEX-corrected EULAR good response, see Additional file 1: Table S3). LUNDEX-corrected EULAR moderate response was reached by 52% at 6 months and by 41% at 12 months (see Additional file 1: Table S3). Twenty-seven percent of patients achieved LUNDEX-corrected HAQ response at 6 months, and 23% did so at 12 months (see Additional file 1: Table S4). Among bionaïve patients, 44% and 46% achieved LUNDEX-corrected EULAR good response at 6 and at 12 months, respectively (see Additional file 1: Table S3). These proportions were significantly higher than in those previously treated with 1 or 2 ≥ bDMARDs (Fig. 2), with similar differences in LUNDEX-corrected HAQ responses (Fig. 3).

There were similar variations for LUNDEX-corrected EULAR moderate response and for proportions attaining LUNDEX-corrected DAS28 remission or low disease activity (see figures in Additional files 2, 3 and 4 and Additional file 1: Tables S3–S5).

### Predictors of EULAR response

Men were more likely than women to achieve a LUNDEX-corrected EULAR good response at 6 months and at 12 months in univariate analysis. These differences also reached statistical significance in the multivariate models (adjusted odds ratio (OR) 2.28, 95% CI 1.45–3.57 and adjusted OR 2.14, 95% CI 1.44–3.19) (Table 3). There were also significant associations between being bionaïve at start of abatacept and a LUNDEX-corrected EULAR good response at 6 months and at 12 months, independently of other predictors [adjusted ORs 3.59 (95% CI 2.25–5.72) at 6 months and 4.29 (95% CI 2.77–6.65) at 12 months] (Table 3). A higher HAQ score at baseline predicted a lower probability of a LUNDEX-corrected EULAR good response at 6 and at 12 months. This negative association between HAQ-DI and clinical response was also significant in the multivariate analysis (Table 3). Age at abatacept start was negatively associated with LUNDEX-corrected EULAR good response at 6 months but not at 12 months. Those on glucocorticoid treatment at baseline were less likely to reach a LUNDEX-corrected EULAR good response at 6 months, but there was no such association at 12 months. Disease duration, abatacept administration route, and treatment with MTX showed some associations with LUNDEX-corrected EULAR good response in the univariate analyses, but not in the multivariate models (Table 3).

**Table 3:** Predictors of LUNDEX-corrected EULAR good response. Odds ratios (95% confidence intervals)

	6 months		12 months	
	Univariate	Multivariate	Univariate	Multivariate
Male sex	2.11 (1.41–3.13)	2.28 (1.45–3.57)	2.46 (1.71–3.54)	2.14 (1.44–3.19)
≥ 2 bDMARDs	Reference (1.0)	Reference (1.0)	Reference (1.0)	Reference (1.0)
Bionaïve	4.00 (2.60–6.15)	3.59 (2.25–5.72)	4.45 (2.95–6.71)	4.29 (2.77–6.65)
1 bDMARD	1.11 (0.72–1.72)	*	1.11 (0.74–1.69)	**
DAS28-CRP (per SD) at baseline	0.95 (0.80–1.14)	*	0.98 (0.83–1.16)	**
DAS28 (per unit) at baseline	0.91 (0.79–1.04)	*	0.97 (0.85–1.10)	**
VAS pain (per SD) at baseline	0.96 (0.80–1.15)	*	0.89 (0.75–1.06)	**
Methotrexate at baseline	1.36 (0.95–1.94)	*	1.46 (1.04–2.06)	**
HAQ score (per SD) at baseline	0.64 (0.52–0.77)	0.75 (0.61–0.93)	0.65 (0.54–0.78)	0.74 (0.61–0.90)
Disease duration (per SD) at baseline	0.79 (0.65–0.96)	*	0.72 (0.59–0.87)	**
Age (per SD) at baseline	0.76 (0.65–0.91)	0.79 (0.65–0.96)	0.97 (0.82–1.14)	**
Glucocorticoids at baseline	0.59 (0.42–0.83)	0.59 (0.40–0.86)	0.72 (0.52–1.00)	**
csDMARD at baseline	1.40 (0.96–2.06)	*	1.25 (0.87–1.79)	**
s.c. abatacept administration	Reference (1.0)	*	Reference (1.0)	**
i.v. abatacept administration	0.53 (0.38–0.75)	*	0.70 (0.51–0.98)	**

*Not included in the final model. The first multivariate model in the stepwise analysis included sex, bDMARD exposure, DAS28, methotrexate at baseline, HAQ-DI, disease duration, age, glucocorticoids at baseline, csDMARDs at baseline, and route of abatacept administration. **Not included in the final model. The first multivariate model in the stepwise analysis included sex, bDMARD exposure, methotrexate at baseline, HAQ-DI, disease duration, glucocorticoids at baseline, and route of abatacept administration. Multivariate model includes 754 patients at 6 months, 829 patients at 12 months

Baseline data for those who were included in the multivariate logistic regression analyses for predictors of Lundex-corrected EULAR good response at 6 and 12 months, and those that were excluded due to missing data for outcome or ≥ 1 of the covariates are listed in Additional file 1: Table S19. Patients with missing data were more likely to receive subcutaneous treatment than intravenous and had slightly lower disease activity, measured by DAS28. Apart from this, there was no major difference between patients with and without missing data for this analysis (see Additional file 1: Table S19).

Biologic DMARD naive patients had higher probability to achieve a LUNDEX-corrected EULAR moderate response at 6 and at 12 months as well. Furthermore, a LUNDEX-corrected EULAR moderate response was predicted in multivariate analysis at 6 months by treatment with a csDMARD at baseline, and at 12 months by male sex (see Additional file 1: Table S6). Results of the sensitivity analyses on predictors of LUNDEX-corrected responses were largely similar to those of the main analyses (see Additional file 1: Tables S7–S18).

### Predictors of HAQ response

Bionaїve patients were more likely than those with ≥ 2 previous bDMARDs to achieve a LUNDEX-corrected HAQ response at 6 and 12 months, independently of other predictors [adjusted OR 2.31 (95% CI 1.49–3.60) and 2.05 (95% CI 1.37–3.07)] (Table 4). HAQ score at baseline showed independent predictive value for LUNDEX-corrected HAQ response at 6 months (adjusted OR 1.73 per SD; 95% CI 1.46–2.05) but not at 12 months (Table 4). Disease duration had a negative association with LUNDEX-corrected HAQ response in the adjusted model at 6 months (adjusted OR 0.74; 95% CI 0.61–0.89) but not at 12 months (Table 4). DAS28, DAS28-CRP, VAS pain, and treatment with MTX at baseline were positively associated with LUNDEX-corrected HAQ response at 6 and 12 months in univariate analyses. However, no predictive value of DAS28, DAS28-CRP, VAS pain, and treatment with MTX was found when adjusting for confounding variables (Table 4).

**Table 4:** Predictors of LUNDEX-corrected HAQ response. Odds ratios (95% confidence intervals)

	6 months		12 months	
	Univariate	Multivariate	Univariate	Multivariate
Male sex	1.07 (0.73–1.58)	*	1.32 (0.91–1.90)	**
≥ 2 bDMARDs	Reference (1.0)	Reference (1.0)	Reference (1.0)	Reference (1.0)
Bionaïve	2.25 (1.50–3.38)	2.31 (1.49–3.60)	2.05 (1.37–3.07)	2.05 (1.37–3.07)
1 bDMARD	1.02 (0.71–1.47)	*	0.70 (0.48–1.04)	**
DAS28-CRP (per SD) at baseline	1.48 (1.26–1.74)	*	1.38 (1.17–1.62)	**
DAS28 (per unit) at baseline	1.40 (1.23–1.60)	*	1.27 (1.12–1.45)	**
VAS pain (per SD) at baseline	1.45 (1.23–1.70)	*	1.24 (1.05–1.47)	**
Methotrexate at baseline	1.50 (1.10–2.05)	*	1.39 (1.01–1.90)	**
HAQ score (per SD) at baseline	1.52 (1.29–1.78)	1.73 (1.46–2.05)	1.24 (1.06–1.45)	**
Disease duration (per SD) at baseline	0.77 (0.65–0.91)	0.74 (0.61–0.89)	0.78 (0.65–0.92)	**
Age (per SD) at baseline	0.93 (0.80–1.08)	*	1.00 (0.86–1.17)	**
Glucocorticoids at baseline	0.75 (0.55–1.01)	*	0.83 (0.61–1.13)	**
csDMARDs at baseline	1.30 (0.94–1.81)	*	1.17 (0.84–1.62)	**
s.c. abatacept administration	Reference (1.0)	*	Reference (1.0)	*
i.v. abatacept administration	0.98 (0.73–1.33)	*	0.97 (0.71–1.32)	**

*Not included in the final model. The first multivariate model in the stepwise analysis included bDMARD exposure, DAS28-CRP, DAS28, VAS pain, methotrexate at baseline, HAQ-DI, disease duration, glucocorticoids at baseline, and csDMARDs at baseline. **Not included in the final model. The first multivariate model in the stepwise analysis included sex, bDMARD exposure, DAS28-CRP, DAS28, VAS pain, methotrexate at baseline, HAQ-DI, and disease duration. Multivariate model includes 862 patients at 6 months and 943 at 12 months

## Discussion

In this study of a large, national cohort, we found that bionaïve patients had a longer survival on abatacept as compared to bDMARD experienced patients. However, multivariate analyses demonstrated that this was to some extent explained by other factors. Female sex and severe pain at treatment start were independently associated with reduced survival on abatacept treatment, and patients on methotrexate were less likely to discontinue abatacept. On the other hand, bionaïve patients had significantly higher likelihood of achieving a clinical and functional response on treatment as compared to bDMARD experienced patients. Lack of previous bDMARD exposure, male sex, and a low HAQ score at treatment start were independent predictors of a good clinical response to abatacept both at 6 and at 12 months. Lack of previous bDMARD exposure was also the only predictor of a good functional response both at 6 and at 12 months.

In observational studies, survival on drug is considered a good indirect and composite measure of effectiveness, safety, and tolerability of a given treatment [23]. While a significant amount of data regarding survival on drug exists for TNF inhibitors, the lack of studies on abatacept has been recognized [24]. In a previous study from the SRQ, drug survival on TNF inhibitors was similar to the present study [25]. By contrast, survival on drug for patients treated with abatacept as their second bDMARD was slightly higher than the overall drug continuation rate in this study (64% compared to 55% at 1 year) [25]. This could be partly due to the different time periods during which the patients were recruited. Frisell et al. included patients starting bDMARDs between 2010 and 2016 whereas our inclusion period was 2006–2017. During the first period after abatacept was licensed for RA treatment in Europe, this drug was mainly used in patients with refractory disease, after failure to different previous bDMARDs and thus with a higher risk of ensuing failure to abatacept too. A further study using data from the SRQ to analyze survival on drug and predictors for discontinuation of tocilizumab in RA patients also reported a drug retention rate of 64% after 1 year [26]. The sample size of that study was smaller (530 patients) in comparison with the present study, and patients with no follow-up data at 1 year were excluded, thus partially accounting for the above differences in outcome.

Studies on data from other registers, focusing on treatment with bDMARDs other than abatacept reported better response and survival on drug in bionaïve patients as compared with bDMARD experienced patients [27–31]. Several studies described the same pattern for abatacept-treated patients [32–34], which is in line with our finding, although we suggest that this may partly be due to confounding by other factors.

With respect to clinical response, there may be a more direct role of previous bDMARD exposure. Better clinical response to abatacept in bionaïve than in bDMARD experienced patients was in accordance with clinical trial findings [22, 35, 36] and with the results from other observational studies [34, 37]. To our knowledge, only one study analyzed the long-term effect of abatacept on function in real life, and, in contrast to our study, the results did not show any difference across bDMARD exposures. However, a greater improvement in HAQ-DI was observed in the bionaïve group after adjusting for age and baseline HAQ-DI [32]. The discrepancy could be explained by different cut-offs for the definition of HAQ response—improvement of ≥ 0.3, in accordance with previous clinical trials [22], in the present study, as opposed to the minimal important difference in HAQ-DI in RA (≥ 0.22) [38, 39] in the other study.

Differences in pain perception could account at least for a part of the observed discrepancy in outcomes between women and men. Several studies found male sex to be a predictor of better treatment response or remission in early RA [40–45]. Results from the Danish DANBIO register showed a predictive value of male sex for treatment response to TNF inhibitors, even though the finding was limited to the cohort of early RA, whereas no difference between male and female patients was observed in established RA [46]. However, in the DANBIO study, the response was categorized as no response vs. EULAR moderate or good response and that could account for the discrepancy with our results. Indeed, our study demonstrated that the strong predictive value of sex for EULAR good response differed from the weaker association with the less stringent EULAR moderate or good response. Data on TNF inhibitors from the British register showed no sex-related differences in EULAR response, but they did show lower DAS28 remission rate for female as compared with male patients [47]. Interestingly, the only observational study that focused on the predictive value of sex in patients treated with abatacept [48] and a study on abatacept and tocilizumab from the Danish register [49] did not show any associations between female sex and treatment response. Again, the less stringent EULAR moderate or good response was used as outcome in these studies. The inconsistencies between the different studies show that the relation between sex and treatment response in RA is far from clear. Of note, sex did not predict HAQ response in the present study.

Our data on the benefit of methotrexate are in line with those from an Australian retrospective cohort [33] and the ACTION study, where association of methotrexate with a longer survival on abatacept was also demonstrated in patients previously exposed to bDMARDs [34]. The predictive value of pain for survival on abatacept was reported in the ACTION study too, with results going in the same direction as in our study [34].

The negative correlation between HAQ-DI at baseline and clinical response was in accordance with data on TNF inhibitors from the British Register [47] and with abatacept data from the Japanese register [50].

Limitations of the present study are related to the observational design and to the large number of patients with missing data, which could impact the value of our results. However, such observational design is considered to be the most appropriate to study the long-term survival on drug and the long-term effect of a drug. We could not take into account anti-citrullinated protein antibody and rheumatoid factor status of our patients, since data on such tests are not included in the register. Furthermore, data on comorbidities, which might influence drug survival and treatment response, were not available. We did not stratify analyses of drug survival by reason for abatacept discontinuation, as data on this in the SRQ have not been validated.

Strengths of this study include the large sample size and the long follow-up. This is one of the largest cohorts worldwide of RA patients treated with abatacept, second only to the Canadian Orencia Response Program (ORP) cohort [32]. The use of LUNDEX correction for the efficacy outcomes allowed to investigate the effect of abatacept combined with tolerability, which is an outcome that is relevant to the patients. As the present study was based on a national quality register with high coverage, the study subjects are representative for patients starting abatacept for RA in Sweden. On the other hand, the results may not apply to patient groups with a different ethnic background, or to study settings with distinct availability of abatacept and other bDMARDs, regulatory guidelines, etc.

## Conclusion

The analysis of one of the largest cohorts of abatacept-treated RA patients gave insights on the differences between bionaïve and bDMARD experienced patients in term of treatment persistency as well as in terms of clinical and functional response. We demonstrated that bionaïve patients had longer survival on abatacept as well as better clinical and functional response to this drug as compared to bDMARD experienced patients. Sex, and to some extent baseline disease severity, also influenced outcome of treatment with abatacept. Insights on the efficacy and tolerability of abatacept based on such data from a real-life setting may be useful for clinical practice.

## Supplementary information

**Additional file 1.** Additional tables. Tables with additional data and analysis.

**Additional file 2 **Proportions of patients achieving LUNDEX corrected EULAR moderate response by previous bDMARD exposure. *p < 0.001 for bionaïve patients vs patients treated with 1 and with ≥2 previous bDMARDs. Bars are 95% CI.

**Additional file 3.** Proportions of patients achieving LUNDEX corrected DAS 28 remission by previous bDMARD exposure. *p < 0.001 for bionaïve patients vs patients treated with 1 and with ≥2 previous bDMARDs. Bars are 95% CI.

**Additional file 4.** Proportions of patients achieving LUNDEX corrected DAS 28 low disease activity by previous bDMARD exposure. *p < 0.001 for bionaïve patients vs patients treated with 1 and with ≥2 previous bDMARDs. Bars are 95% CI.

## Data Availability

The dataset used and analyzed during the current study is available from the corresponding author on reasonable request.

## Abbreviations

bDMARDs: Biologic disease-modifying antirheumatic drugs
CI: Confidence interval
csDMARD: Conventional synthetic disease-modifying antirheumatic drugs
DAS28: Disease activity score on 28 joints
DMARDs: Disease-modifying antirheumatic drugs
HAQ: Health Assessment Questionnaire
HAQ-DI: Health Assessment Questionnaire Disability Index
HR: Hazard ratio
MTX: Methotrexate
OR: Odds ratio
RA: Rheumatoid arthritis
SRQ: Swedish Rheumatology Quality Register
TNF: Tumor necrosis factor
VAS: Visual analog scale
