# Supplementary material for: Abatacept in rheumatoid arthritis: survival on drug, clinical outcomes, and their predictors—data from a large national quality register
Source: Arthritis Res Ther. 2020 Jan 22;22:15. doi: 10.1186/s13075-020-2100-y (PMC6977240; doi:10.1186/s13075-020-2100-y)
Supplement: Supplementary file 1 — Additional file 1. Additional tables. Tables with additional data and analysis. [file 13075_2020_2100_MOESM1_ESM.docx]

Table S1. Sensitivity analysis. Predictors of abatacept discontinuation. Cox regression analysis. Hazard ratios (95% confidence intervals).

|  | Multivariate Analysis (model 1) | Multivariate Analysis (model 2) |
| --- | --- | --- |
| Male sex | 0.84 (0.72-0.97) | 0.86 (0.75-1.00) |
| ≥2 bDMARDs | reference (1.0) | reference (1.0) |
| Bionaïve | 0.88 (0.73-1.06) | 0.91 (0.77-1.08) |
| 1 bDMARDs | 1.02 (0.89-1.18) | 1.04 (0.92-1.19) |
| DAS28 CRP (per SD) at baseline | 1.06 (0.99-1.14) | * |
| VAS pain (per SD) at baseline | 1.09 (1.01-1.18) | 1.13 (1.07-1.20) |
| Methotrexate at baseline | 0.95 (0.77-1.18) | 0.83 (0.74-0.93) |
| HAQ score (per SD) at baseline | 1.01 (0.94-1.09) | * |
| Disease duration (per SD) | 0.94 (0.88-1.01) | 0.94 (0.89-1.00) |
| Age (per SD) at baseline | 0.99 (0.92-1.06) | * |
| Glucocorticoids at baseline | 1.04 (0.92-1.18) | 1.00 (0.89-1.13) |
| csDMARDs at baseline | 0.88 (0.71-1.11) | * |
| sc Abatacept | reference (1.0) | reference (1.0) |
| iv Abatacept | 1.03 (0.90-1.16) | 1.04 (0.92-1.17) |

*= Not included in the final model due to collinearity.

Table S2. Sensitivity analysis. Predictors of abatacept discontinuation. Cox regression analysis. Covariates with p<0.1 in the univariate analysis. Hazard ratios. (95% confidence intervals).

|  | Multivariate Analysis (model 1) | Multivariate Analysis (model 2) |
| --- | --- | --- |
| Male sex | 0.84 (0.72-0.97) | 0.86 (0.75-0.99) |
| ≥2 bDMARDs | reference (1.0) | reference (1.0) |
| Bionaïve | 0.89 (0.75-1.06) | 0.92 (0.78-1.09) |
| 1 bDMARDs | 1.02 (0.89-1.16) | 1.04 (0.92-1.19) |
| DAS28 CRP (per SD) at baseline | 1.07 (1.00-1.15) | * |
| VAS pain (per SD) at baseline | 1.10 (1.02-1.18) | 1.14 (1.07-1.20) |
| Methotrexate at baseline | 0.88 (0.78-1.99) | 0.85 (0.75-0.95) |
| HAQ score (per SD) at baseline | 1.00 (0.93-1.07) | * |

* Not included in the final model due to collinearity.

Pearson´s correlation:

Age (per SD) * Disease duration (per SD): 0.32

DAS28 CRP (per SD) * HAQ score (per SD): 0.44

DAS28 CRP (per SD) * VAS pain (per SD): 0.52

VAS pain (per SD) * HAQ score (per SD): 0.52

Spearman´s correlation:

Methotrexate * csDMARDs: 0.83

Table S3. EULAR response and LUNDEX corrected EULAR response at 6 and 12 months.

|  | 6 months | | | | 12 months | | | |
| --- | --- | --- | --- | --- | --- | --- | --- | --- |
|  | Total | Bionaïve | 1 bDMARD | ≥2 bDMARDs | Total | Bionaïve | 1 bDMARD | ≥2 bDMARDs |
| Patients | 789 | 125 | 201 | 463 | 872 | 136 | 225 | 511 |
| EULAR Good response | 189 (24%) | 56 (45%) | 42 (21%) | 91 (20%) | 254 (29%) | 72 (53%) | 53 (24%) | 129 (25%) |
| EULAR Moderate response | 466 (59%) | 99 (79%) | 117 (58%) | 250 (54%) | 540 (62%) | 100 (73%) | 134 (60%) | 306 (60%) |
| LUNDEX EULAR Good response | 167 (21%) | 55 (44%) | 36 (18%) | 76 (16%) | 186 (21%) | 63 (46%) | 40 (18%) | 83 (16%) |
| LUNDEX EULAR Moderate response | 409 (52%) | 95 (76%) | 98 (49%) | 216 (47%) | 359 (41%) | 82 (60%) | 89 (40%) | 188 (37%) |

Table S4. HAQ response and LUNDEX corrected HAQ response at 6 and at 12 months.

|  | 6 months | | | | 12 months | | | |
| --- | --- | --- | --- | --- | --- | --- | --- | --- |
|  | Total | Bionaïve | 1 bDMARD | ≥2 bDMARDs | Total | Bionaïve | 1 bDMARD | ≥2 bDMARDs |
| Patients | 864 | 127 | 223 | 514 | 943 | 133 | 246 | 564 |
| HAQ response | 267 (31%) | 60 (47%) | 62 (28%) | 145 (28%) | 307 (33%) | 56 (42%) | 58 (24%) | 193 (34%) |
| LUNDEX HAQ response | 237 (27%) | 54 (42%) | 56 (25%) | 127 (25%) | 215 (23%) | 49 (37%) | 41 (17%) | 125 (22%) |

Table S5. DAS 28 response and LUNDEX corrected DAS 28 response at 6 and at 12 months.

|  | 6 months | | | | 12 months | | | |
| --- | --- | --- | --- | --- | --- | --- | --- | --- |
|  | Total | Bionaïve | 1 bDMARD | ≥2 bDMARDs | Total | Bionaïve | 1 bDMARD | ≥2 bDMARDs |
| Patients | 994 | 156 | 262 | 576 | 1117 | 172 | 289 | 656 |
| Low disease activity | 319 (32%) | 83 (53%) | 80 (30%) | 156 (27%) | 402 (36%) | 98 (57%) | 92 (32%) | 212 (32%) |
| Remission | 172 (17%) | 51 (33%) | 45 (17%) | 76 (13%) | 239 (21%) | 69 (40%) | 43 (15%) | 127 (19%) |
| LUNDEX Low disease activity | 275 (28%) | 79 (51%) | 68 (26%) | 128 (22%) | 291 (26%) | 85 (49%) | 68 (24%) | 138 (21%) |
| LUNDEX remission | 151 (15%) | 49 (31%) | 38 (15%) | 64 (11%) | 175 (16%) | 59 (34%) | 34 (12%) | 82 (13%) |

Table S6. Predictors of LUNDEX corrected EULAR moderate response at 6 and 12 months. Odds ratios (95% confidence intervals)

|  | 6 months | 12 months |
| --- | --- | --- |
| Male sex | * | 1.70 (1.21-2.38) |
| ≥2 bDMARDs | reference (1.0) | reference (1.0) |
| Bionaïve | 3.73 (2.37-5.86) | 2.54 (1.72-3.75) |
| DAS 28 CRP (per SD) | * | ** |
| DAS 28 (per unit) | * | ** |
| DAS 28 (per SD) | * | ** |
| VAS pain (per SD) | * | ** |
| Methotrexate at baseline | * | ** |
| HAQ score (per SD) | * | ** |
| Disease duration (per SD) | * | ** |
| Glucocorticoids at baseline | * | ** |
| csDMARDs at baseline | 1.50 (1.10-2.05) | ** |

* Not included in the final model. The first multivariate model in the stepwise analysis included: bDMARD exposure, DAS28 CRP, DAS28, VAS pain, Methotrexate at baseline, disease duration, csDMARDs at baseline. ** Not included in the final model. The first multivariate model in the stepwise analysis included: sex, bDMARD exposure, DAS28 CRP, DAS28, Methotrexate at baseline, HAQ-DI, disease duration, glucocorticoids at baseline, csDMARDs at baseline. Multivariate model includes 789 patients at 6 months and 872 patients at 12 months.

Table S7. Sensitivity analysis. Predictors of LUNDEX corrected EULAR Good Response at 6 months. Logistic regression analysis. Odds ratios (95% confidence intervals).

|  | Multivariate Analysis (model 1) | Multivariate Analysis (model 2) |
| --- | --- | --- |
| Male sex | 2.13 (1.34-3.40) | 2.28 (1.45-3.58) |
| ≥2 bDMARDs | reference (1.0) | reference (1.0) |
| Bionaïve | 4.03 (2.38-6.83) | 3.51 (2.16-5.69) |
| 1 bDMARDs | 1.16 (0.71-1.90) | 1.06 (0.66-1.71) |
| DAS28 CRP (per SD) at baseline | 2.01 (1.16-3.49) | * |
| VAS pain (per SD) at baseline | 1.10 (0.86-1.41) | * |
| Methotrexate at baseline | 1.08 (0.55-2.11) | * |
| HAQ score (per SD) at baseline | 0.68 (0.53-0.88) | 0.76 (0.61-0.94) |
| Disease duration (per SD) | 1.14 (0.91-1.44) | * |
| Age (per SD) at baseline | 0.80 (0.65-0.99) | 0.80 (0.65-0.97) |
| Glucocorticoids at baseline | 0.62 (0.42-0.91) | 0.62 (0.42-0.92) |
| csDMARDs at baseline | 1.35 (0.66-2.79) | 1.42 (0.93-2.17) |
| sc Abatacept | reference (1.0) | reference (1.0) |
| iv Abatacept | 0.73 (0.49-1.11) | 0.78 (0.52-1.15) |
| DAS28 (per unit) | 0.62 (0.41-0.94) | * |

* Not included in the final model due to collinearity.

Pearson´s correlation:

Age (per SD) * Disease duration (per SD): 0.32

DAS28 CRP (per SD) * HAQ score (per SD): 0.44

DAS28 CRP (per SD) * VAS pain (per SD): 0.52

DAS 28 (per unit) * DAS28 CRP (per SD): 0.92

DAS 28 (per unit) * VAS pain (per SD): 0.47

DAS 28 (per unit) * HAQ score (per SD): 0.44

VAS pain (per SD) * HAQ score (per SD): 0.52

Spearman´s correlation:

Methotrexate * csDMARDs: 0.83

Table S8. Sensitivity analysis. Predictors of LUNDEX corrected EULAR Good Response at 6 months. Logistic regression analysis. Covariates with p<0.1 in the univariate analysis. Odds ratios (95% confidence intervals).

|  | Multivariate Analysis (model 1) | Multivariate Analysis (model 2) |
| --- | --- | --- |
| Male sex | 2.10 (1.35-3.26) | 2.28 (1.45-3.57) |
| ≥2 bDMARDs | reference (1.0) | reference (1.0) |
| Bionaïve (2 bDMARDs ref.) | 3.35 (2.12-5.29) | 3.59 (2.25-5.72) |
| 1 bDMARDs (2 bDMARDs ref.) | 1.00 (0.63-1.60) | 1.08 (0.67-1.74) |
| DAS28 CRP (per SD) at baseline | * | * |
| VAS pain (per SD) at baseline | * | * |
| Methotrexate at baseline | * | ** |
| HAQ score (per SD) at baseline | 0.70 (0.57-0.86) | 0.75 (0.61-0.93) |
| Disease duration (per SD) | * | ** |
| Age (per SD) at baseline | * | 0.79 (0.65-0.96) |
| Glucocorticoids at baseline | 0.57 (0.39-0.84) | 0.59 (0.40-0.86) |
| csDMARDs at baseline | * | * |
| sc Abatacept | * | * |
| iv Abatacept (sc ABA=ref.) | * | * |
| DAS 28 (per unit) | * | ** |

* Not included in the final model. ** Not included in the final model due to collinearity.

Table S9. Sensitivity analysis. Predictors of LUNDEX corrected EULAR Good Response at 12 months. Logistic regression analysis. Odds ratios (95% confidence intervals).

|  | Multivariate Analysis (model 1) | Multivariate Analysis (model 2) |
| --- | --- | --- |
| Male sex | 2.04 (1.33-3.12) | 2.15 (1.42-3.25) |
| ≥2 bDMARDs | reference (1.0) | reference (1.0) |
| Bionaïve | 4.13 (2.55-6.68) | 4.02 (2.53-6.39) |
| 1 bDMARDs | 1.09 (0.69-1.73) | 1.02 (0.65-1.60) |
| DAS28 CRP (per SD) at baseline | 1.49 (0.91-2.46) | * |
| VAS pain (per SD) at baseline | 1.02 (0.81-1.30) | * |
| Methotrexate at baseline | 1.88 (0.92-3.83) | 1.36 (0.93-1.99) |
| HAQ score (per SD) at baseline | 0.67 (0.52-0.86) | 0.76 (0.62-0.93) |
| Disease duration (per SD) | 0.97 (0.78-1.21) | 0.94 (0.77-1.15) |
| Age (per SD) at baseline | 1.02 (0.83-1.26) | * |
| Glucocorticoids at baseline | 0.76 (0.52-1.10) | 0.76 (0.52-1.11) |
| csDMARDs at baseline | 0.66 (0.31-1.41) | * |
| sc Abatacept | reference (1.0) | reference (1.0) |
| iv Abatacept | 0.80 (0.54-1.16) | 0.84 (0.58-1.21) |
| DAS28 (per unit) | 0.83 (0.57-1.21) | * |

* Not included in the final model due to collinearity.

Table S10. Sensitivity analysis. Predictors of LUNDEX corrected EULAR Good Response at 12 months. Logistic regression analysis. Covariates with p<0.1 in the univariate analysis. Odds ratios (95% confidence intervals).

|  | Multivariate Analysis (model 1) | Multivariate Analysis (model 2) |
| --- | --- | --- |
| Male sex | 2.14 (1.44-3.19) | 2.14 (1.44-3.19) |
| ≥2 bDMARDs | reference (1.0) | reference (1.0) |
| Bionaïve | 4.29 (2.77-6.65) | 4.29 (2.77-6.65) |
| 1 bDMARDs | 1.12 (0.72-1.73) | 1.12 (0.72-1.73) |
| DAS28 CRP (per SD) at baseline | * | ** |
| VAS pain (per SD) at baseline | * | ** |
| Methotrexate at baseline | * | * |
| HAQ score (per SD) at baseline | 0.74 (0.61-0.90) | 0.74 (0.61-0.90) |
| Disease duration (per SD) | * | * |
| Age (per SD) at baseline | * | ** |
| Glucocorticoids at baseline | * | * |
| csDMARDs at baseline | * | ** |
| sc Abatacept | * | * |
| iv Abatacept (sc ABA=ref.) | * | * |
| DAS 28 (per unit) | * | ** |

* Not included in the final model. ** Not included in the final model due to collinearity.

Table S11. Sensitivity analysis. Predictors of LUNDEX corrected EULAR Moderate response at 6 months. Logistic regression analysis. Odds ratios (95% confidence intervals).

|  | Multivariate Analysis (model 1) | Multivariate Analysis (model 2) |
| --- | --- | --- |
| Male sex | 1.08 (0.72-1.62) | 1.17 (0.79-1.72) |
| ≥2 bDMARDs | reference (1.0) | reference (1.0) |
| Bionaïve | 3.66 (2.21-6.09) | 3.95 (2.42-6.44) |
| 1 bDMARDs | 1.05 (0.73-1.51) | 1.12 (0.79-1.58) |
| DAS28 CRP (per SD) at baseline | 1.01 (0.65-1.58) | 1.38 (1.17-1.61) |
| VAS pain (per SD) at baseline | 1.01 (0.84-1.23) | * |
| Methotrexate at baseline | 1.02 (0.59-1.75) | * |
| HAQ score (per SD) at baseline | 0.84 (0.69-1.03) | * |
| Disease duration (per SD) | 1.07 (0.89-1.28) | 1.02 (0.87-1.19) |
| Age (per SD) at baseline | 1.03 (0.87-1.23) | * |
| Glucocorticoids at baseline | 0.88 (0.64-1.20) | 0.90 (0.67-1.22) |
| csDMARDs at baseline | 1.44 (0.81-2.55) | 1.43 (1.04-1.97) |
| sc Abatacept | reference (1.0) | reference (1.0) |
| iv Abatacept | 1.10 (0.79-1.53) | 1.11 (0.81-1.53) |
| DAS28 (per unit) | 1.36 (0.97-1.89) | * |

* Not included in the final model due to collinearity.

Table S12. Sensitivity analysis. Predictors of LUNDEX corrected EULAR Moderate Response at 6 months. Covariates with p<0.1 in the univariate analysis. Logistic regression analysis. Odds ratios (95% confidence intervals).

|  | Multivariate Analysis (model 1) | Multivariate Analysis (model 2) |
| --- | --- | --- |
| Male sex | * | * |
| ≥2 bDMARDs | reference (1.0) | reference (1.0) |
| Bionaïve (2 bDMARDs ref.) | 3.80 (2.35-6.13) | 3.96 (2.50-6.27) |
| 1 bDMARDs (2 bDMARDs ref.) | 1.04 (0.73-1.47) | 1.12 (0.79-1.57) |
| DAS28 CRP (per SD) at baseline | * | 1.38 (1.19-1.61) |
| VAS pain (per SD) at baseline | * | ** |
| Methotrexate at baseline | * | ** |
| HAQ score (per SD) at baseline | * | ** |
| Disease duration (per SD) | * | * |
| Age (per SD) at baseline | * | ** |
| Glucocorticoids at baseline | * | * |
| csDMARDs at baseline | * | 1.46 (1.07-2.01) |
| sc Abatacept | * | * |
| iv Abatacept | * | * |
| DAS 28 (per unit) | * | ** |

* Not included in the final model. ** Not included in the final model due to collinearity.

Table S13. Sensitivity analysis. Predictors of LUNDEX corrected EULAR Moderate Response at 12 months. Logistic regression analysis. Odds ratios (95% confidence intervals).

|  | Multivariate Analysis (model 1) | Multivariate Analysis (model 2) |
| --- | --- | --- |
| Male sex | 1.52 (1.05-2.21) | 1.66 (1.17-2.35) |
| ≥2 bDMARDs | reference (1.0) | reference (1.0) |
| Bionaïve | 2.60 (1.67-4.04) | 2.70 (1.78-4.11) |
| 1 bDMARDs | 1.12 (0.79-1.60) | 1.12 (0.80-1.56) |
| DAS28 CRP (per SD) at baseline | 1.28 (0.85-1.93) | 1.31 (1.13-1.52) |
| VAS pain (per SD) at baseline | 0.91 (0.76-1.11) | * |
| Methotrexate at baseline | 1.02 (0.62-1.70) | * |
| HAQ score (per SD) at baseline | 0.78 (0.64-0-95) | * |
| Disease duration (per SD) | 1.08 (0.91-1.28) | 1.00 (0.86-1.16) |
| Age (per SD) at baseline | 0.97 (0.82-1.15) | * |
| Glucocorticoids at baseline | 0.79 (0.58-1.07) | 0.79 (0.59-1.06) |
| csDMARDs at baseline | 1.29 (0.75-2.22) | 1.41 (1.03-1.93) |
| sc Abatacept | reference (1.0) | reference (1.0) |
| iv Abatacept | 1.12 (0.82-1.52) | 1.14 (0.84-1.53) |
| DAS28 (per unit) | 1.18 (0.86-1.61) | * |

* Not included in the final model due to collinearity.

Table S14. Sensitivity analysis. Predictors of LUNDEX corrected EULAR Moderate Response at 12 months. Logistic regression analysis. Covariates with p<0.1 in the univariate analysis. Odds ratios (95% confidence intervals).

|  | Multivariate Analysis (model 1) | Multivariate Analysis (model 2) |
| --- | --- | --- |
| Male sex | * | 1.63 (1.16-2.29) |
| ≥2 bDMARDs | reference (1.0) | reference (1.0) |
| Bionaïve | 2.51 (1.68-3.76) | 2.73 (1.83-4.05) |
| 1 bDMARDs | 1.12 (0.80-1.56) | 1.16 (0.83-1.61) |
| DAS28 CRP (per SD) at baseline | * | 1.32 (1.14-1.53) |
| VAS pain (per SD) at baseline | * | ** |
| Methotrexate at baseline | * | ** |
| HAQ score (per SD) at baseline | * | ** |
| Disease duration (per SD) | * | * |
| Age (per SD) at baseline | * | ** |
| Glucocorticoids at baseline | * | * |
| csDMARDs at baseline | * | 1.44 (1.06-1.96) |
| sc Abatacept | * | * |
| iv Abatacept (sc ABA=ref.) | * | * |
| DAS 28 (per unit) | * | ** |

* Not included in the final model.

** Not included in the final model due to collinearity.

Table S15. Sensitivity analysis. Predictors of LUNDEX corrected HAQ Response at 6 months. Logistic regression analysis. Odds ratios (95% confidence intervals).

|  | Multivariate Analysis (model 1) | Multivariate Analysis (model 2) |
| --- | --- | --- |
| Male sex | 1.16 (0.74-1.82) | 1.16 (0.77-1.76) |
| ≥2 bDMARDs | reference (1.0) | reference (1.0) |
| Bionaïve | 2.65 (1.60-4.39) | 2.33 (1.46-3.69) |
| 1 bDMARDs | 1.14 (0.76-1.72) | 0.96 (0.66-1.40) |
| DAS28 CRP (per SD) at baseline | 1.11 (0.67-1.83) | * |
| VAS pain (per SD) at baseline | 1.06 (0.86-1.32) | * |
| Methotrexate at baseline | 1.73 (0.89-3.38) | 1.52 (1.09-2.12) |
| HAQ score (per SD) at baseline | 1.57 (1.26-1.95) | 1.75 (1.47-2.08) |
| Disease duration (per SD) | 0.83 (0.67-1.02) | 0.76 (0.63-0.92) |
| Age (per SD) at baseline | 0.85 (0.71-1.02) | * |
| Glucocorticoids at baseline | 0.80 (0.57-1.14) | 0.79 (0.57-1.09) |
| csDMARDs at baseline | 0.81 (0.40-1.63) | * |
| sc Abatacept | reference (1.0) | reference (1.0) |
| iv Abatacept | 0.93 (0.64-1.34) | 0.95 (0.68-1.33) |
| DAS 28 (per unit) | 1.10 (0.75-1.61) | * |

* Not included in the final model due to collinearity.

Table S16. Sensitivity analysis. Predictors of LUNDEX corrected HAQ Response at 6 months. Logistic regression analysis. Covariates with p<0.1 in the univariate analysis. Odds ratios (95% confidence intervals).

|  | Multivariate Analysis (model 1) | Multivariate Analysis (model 2) |
| --- | --- | --- |
| Male sex | * | * |
| ≥2 bDMARDs | reference (1.0) | reference (1.0) |
| Bionaïve | 2.46 (1.57-3.85) | 2.46 (1.57-3.85) |
| 1 bDMARDs | 0.97 (0.66-1.41) | 0.97 (0.66-1.41) |
| DAS28 CRP (per SD) at baseline | * | ** |
| VAS pain (per SD) at baseline | * | ** |
| Methotrexate at baseline | 1.56 (1.12-2.17) | 1.56 (1.12-2.17) |
| HAQ score (per SD) at baseline | 1.73 (1.46-2.05) | 1.73 (1.46-2.05) |
| Disease duration (per SD) | 0.76 (0.63-0.91) | 0.76 (0.63-0.91) |
| Age (per SD) at baseline | * | ** |
| Glucocorticoids at baseline | * | * |
| csDMARDs at baseline | * | ** |
| sc Abatacept | * | * |
| iv Abatacept | * | * |
| DAS 28 (per unit) | * | ** |

* Not included in the final model. ** Not included in the final model due to collinearity.

Table S17. Sensitivity analysis. Predictors of LUNDEX corrected HAQ Response at 12 months. Logistic regression analysis. Odds ratios (95% confidence intervals).

|  | Multivariate Analysis (model 1) | Multivariate Analysis (model 2) |
| --- | --- | --- |
| Male sex | 1.26 (0.83-1.91) | 1.20 (0.81-1.78) |
| ≥2 bDMARDs | reference (1.0) | reference (1.0) |
| Bionaïve | 1.97 (1.24-3.15) | 1.97 (1.27-3.07) |
| 1 bDMARDs | 0.69 (0.45-1.06) | 0.76 (0.51-1.14) |
| DAS28 CRP (per SD) at baseline | 1.16 (0.72-1.85) | 1.41 (1.19-1.67) |
| VAS pain (per SD) at baseline | 1.00 (0.80-1.25) | * |
| Methotrexate at baseline | 1.83 (0.95-3.56) | 1.35 (0.96-1.88) |
| HAQ score (per SD) at baseline | 1.21 (0.97-1.51) | * |
| Disease duration (per SD) | 0.81 (0.66-0.99) | 0.87 (0.73-1.04) |
| Age (per SD) at baseline | 1.08 (0.89-1.31) | * |
| Glucocorticoids at baseline | 0.79 (0.56-1.12) | 0.79 (0.57-1.10) |
| csDMARDs at baseline | 0.66 (0.33-1.34) | * |
| sc Abatacept | reference (1.0) | reference (1.0) |
| iv Abatacept | 0.86 (0.61-1.22) | 0.87 (0.62-1.22) |
| DAS 28 (per unit) | 1.07 (0.75-1.53) | * |

* Not included in the final model due to collinearity.

Table S18. Sensitivity analysis. Predictors of LUNDEX corrected HAQ Response at 12 months. Logistic regression analysis. Covariates with p<0.1 in the univariate analysis. Odds ratios (95% confidence intervals).

|  | Multivariate Analysis (model 1) | Multivariate Analysis (model 2) |
| --- | --- | --- |
| Male sex | * | * |
| ≥2 bDMARDs | reference (1.0) | reference (1.0) |
| Bionaïve | 2.22 (1.42-3.47) | 2.31 (1.53-3.50) |
| 1 bDMARDs | 0.71 (0.47-1.09) | 0.80 (0.54-1.19) |
| DAS28 CRP (per SD) at baseline | * | 1.40 (1.18-1.65) |
| VAS pain (per SD) at baseline | * | ** |
| Methotrexate at baseline | * | * |
| HAQ score (per SD) at baseline | * | ** |
| Disease duration (per SD) | * | * |
| Age (per SD) at baseline | * | ** |
| Glucocorticoids at baseline | * | * |
| csDMARDs at baseline | * | ** |
| sc Abatacept | * | * |
| iv Abatacept | * | * |
| DAS 28 (per unit) | * | ** |

* Not included in the final model. ** Not included in the final model due to collinearity.

Table S19. Characteristics of those included in the multivariate logistic regression analyses for predictors of Lundex corrected EULAR good response at 6 and 12 months, and those that were excluded due to missing data for outcome or ≥1 of the covariates.

|  | 6 months | | 12 months | |
| --- | --- | --- | --- | --- |
|  | Non missing | Missing | Non missing | Missing |
| Number of patients (%) | 754 | 1962 | 829 | 1887 |
| Female sex (%) | 614 (81.4) | 1562 (79.6) | 660 (79.6) | 1516 (80.3%) |
| Age at treatment start  (years); mean (SD) | 58 (13.0) | 59.7 (13.3) | 58.4 (13.0) | 59.6 (13.4) |
| Duration of RA at treatment start  (years); mean (SD) | 13.1 (10.9) | 14.6 (11.5) | 13.6 (11.1) | 14.5 (11.5) |
| Intravenous treatment | 447 (59.3%) | 918 (46.8%) | 482 (58.1%) | 883 (46.8%) |
| Subcutaneous treatment | 303 (40.2%) | 1035 (52.8%) | 343 (41.4%) | 995 (52.7%) |
| SR (mm 1^st^h); median (IQR) | 23 (11-42) | 23 (11-41) | 23 (11-42) | 23 (11-42) |
| CRP (mg/l); median (IQR) | 9 (3-23) | 9 (3.9-23) | 9 (3-25) | 8 (4-21.8) |
| DAS28; mean (SD) | 5.11 (1.3) | 4.88 (1.3) | 5.09 (1.27) | 4.88 (1.30) |
| DAS28-CRP; mean (SD) | 4.8 (1.1) | 4.6 (1.1) | 4.80 (1.12) | 4.55 (1.13) |
| VAS pain (0-100); mean (SD) | 60.9 (22.3) | 59.9 (23.2) | 61.7 (21.2) | 59.3 (23.89) |
| VAS global (0-100); mean (SD) | 60.5 (22.4) | 60.5 (22.5) | 61.5 (21.7) | 59.8 (23) |
| Swollen joint count (0-28); median (IQR) | 6 (3-10) | 5 (2-8) | 5 (2-8) | 5 (2-8) |
| Tender joint count (0-28); median (IQR) | 7 (4-12) | 6 (3-10) | 6 (3-10) | 6 (3-10) |
| HAQ-DI (0-3); median (IQR) | 1.25 (0.88-1.75) | 1.25 (0.88-1.75) | 1.25 (0.88-1.75) | 1.25 (0.88-1.75) |
| Physicians global (0-4); median (IQR) | 2 (2-3) | 2 (2-3) | 2 (2-3) | 2 (2-3) |
| Current MTX | 445 (59%) | 843 (55.4%) | 490 (59.1%) | 798 (42.3%) |
| Current glucocorticosteroid | 428 (56.8%) | 888 (45.3%) | 499 (60.2%) | 817 (43.3%) |
| Current csDMARD | 512 (67.9%) | 977 (49.8%) | 567 (68.4%) | 922 (48.9%) |
| Bionaive | 119 (15.8%) | 334 (17%) | 128 (15.4%) | 325 (17.2%) |
| 1 previous bDMARD | 188 (24.9%) | 553 (28.2%) | 212 (25.6%) | 529 (28%) |
| ≥ 2 previous bDMARDs | 447 (59.3%) | 1075 (54.8%) | 489 (59%) | 1033 (54.7%) |
