# Supplementary material for: Abatacept in rheumatoid arthritis: survival on drug, clinical outcomes, and their predictors—data from a large national quality register
Source: Arthritis Res Ther. 2020 Jan 22;22:15. doi: 10.1186/s13075-020-2100-y (PMC6977240; doi:10.1186/s13075-020-2100-y)
Supplement: Supplementary file 2 — Additional file 2 Proportions of patients achieving LUNDEX corrected EULAR moderate response by previous bDMARD exposure. *p < 0.001 for bionaïve patients vs patients treated with 1 and with ≥2 previous bDMARDs. Bars are 95% CI. [file 13075_2020_2100_MOESM2_ESM.pptx]

## Slide 1
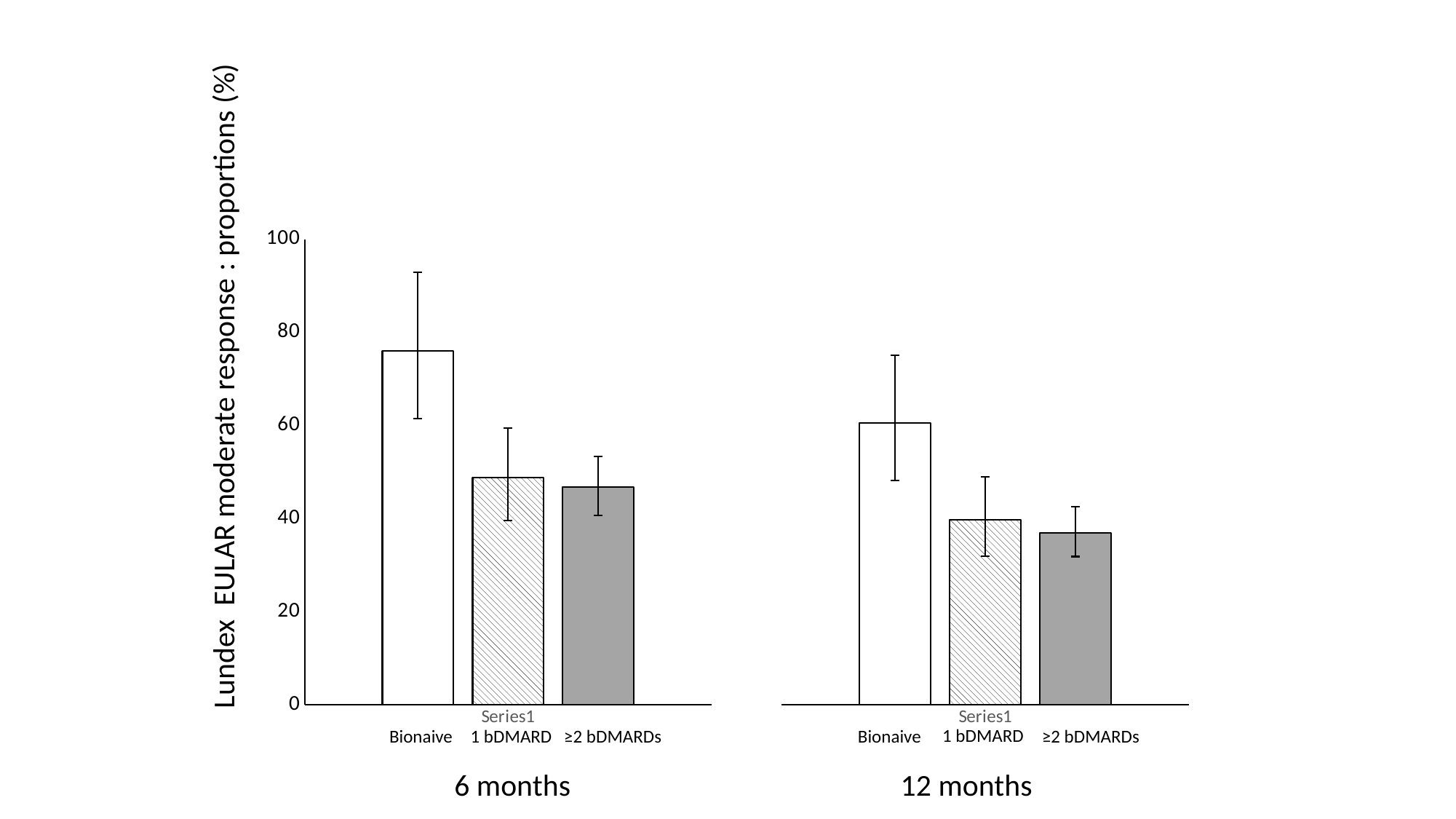

Lundex EULAR moderate response : proportions (%)
### Chart
| Category | Serie 1 | Serie 2 | Serie 3 |
|---|---|---|---|
| | 76.0 | 48.8 | 46.7 |
### Chart
| Category | Serie 1 | Serie 2 | Serie 3 |
|---|---|---|---|
| | 60.3 | 39.6 | 36.8 |1 bDMARD
Bionaive
Bionaive
≥2 bDMARDs
≥2 bDMARDs
1 bDMARD
6 months
12 months
