# Supplementary material for: Abatacept in rheumatoid arthritis: survival on drug, clinical outcomes, and their predictors—data from a large national quality register
Source: Arthritis Res Ther. 2020 Jan 22;22:15. doi: 10.1186/s13075-020-2100-y (PMC6977240; doi:10.1186/s13075-020-2100-y)
Supplement: Supplementary file 3 — Additional file 3. Proportions of patients achieving LUNDEX corrected DAS 28 remission by previous bDMARD exposure. *p < 0.001 for bionaïve patients vs patients treated with 1 and with ≥2 previous bDMARDs. Bars are 95% CI. [file 13075_2020_2100_MOESM3_ESM.pptx]

## Slide 1
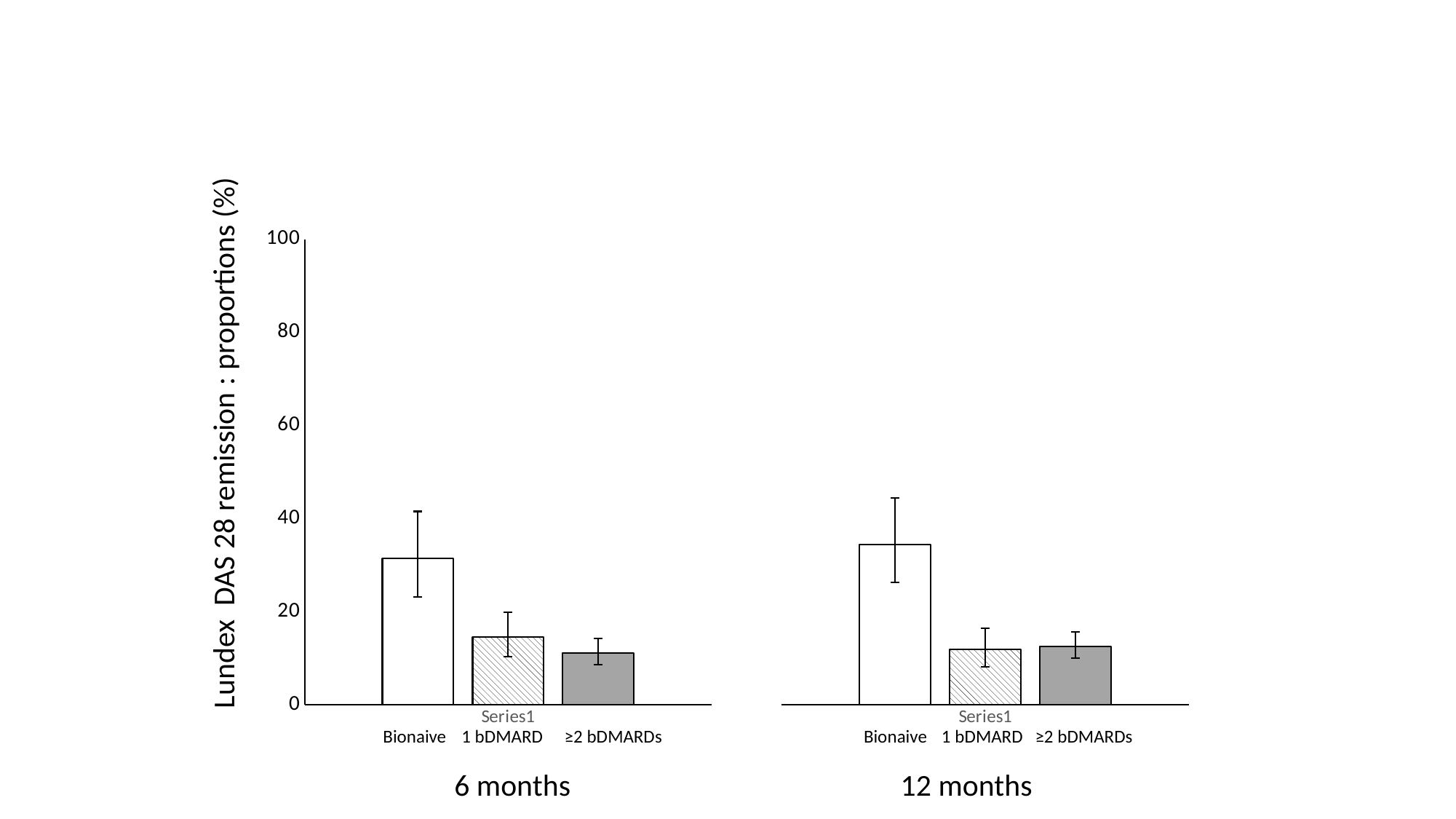

Lundex DAS 28 remission : proportions (%)
### Chart
| Category | Serie 1 | Serie 2 | Serie 3 |
|---|---|---|---|
| | 31.4 | 14.5 | 11.1 |
### Chart
| Category | Serie 1 | Serie 2 | Serie 3 |
|---|---|---|---|
| | 34.3 | 11.8 | 12.5 |Bionaive
≥2 bDMARDs
≥2 bDMARDs
1 bDMARD
Bionaive
1 bDMARD
6 months
12 months
