# Supplementary material for: Abatacept in rheumatoid arthritis: survival on drug, clinical outcomes, and their predictors—data from a large national quality register
Source: Arthritis Res Ther. 2020 Jan 22;22:15. doi: 10.1186/s13075-020-2100-y (PMC6977240; doi:10.1186/s13075-020-2100-y)
Supplement: Supplementary file 4 — Additional file 4. Proportions of patients achieving LUNDEX corrected DAS 28 low disease activity by previous bDMARD exposure. *p < 0.001 for bionaïve patients vs patients treated with 1 and with ≥2 previous bDMARDs. Bars are 95% CI. [file 13075_2020_2100_MOESM4_ESM.pptx]

## Slide 1
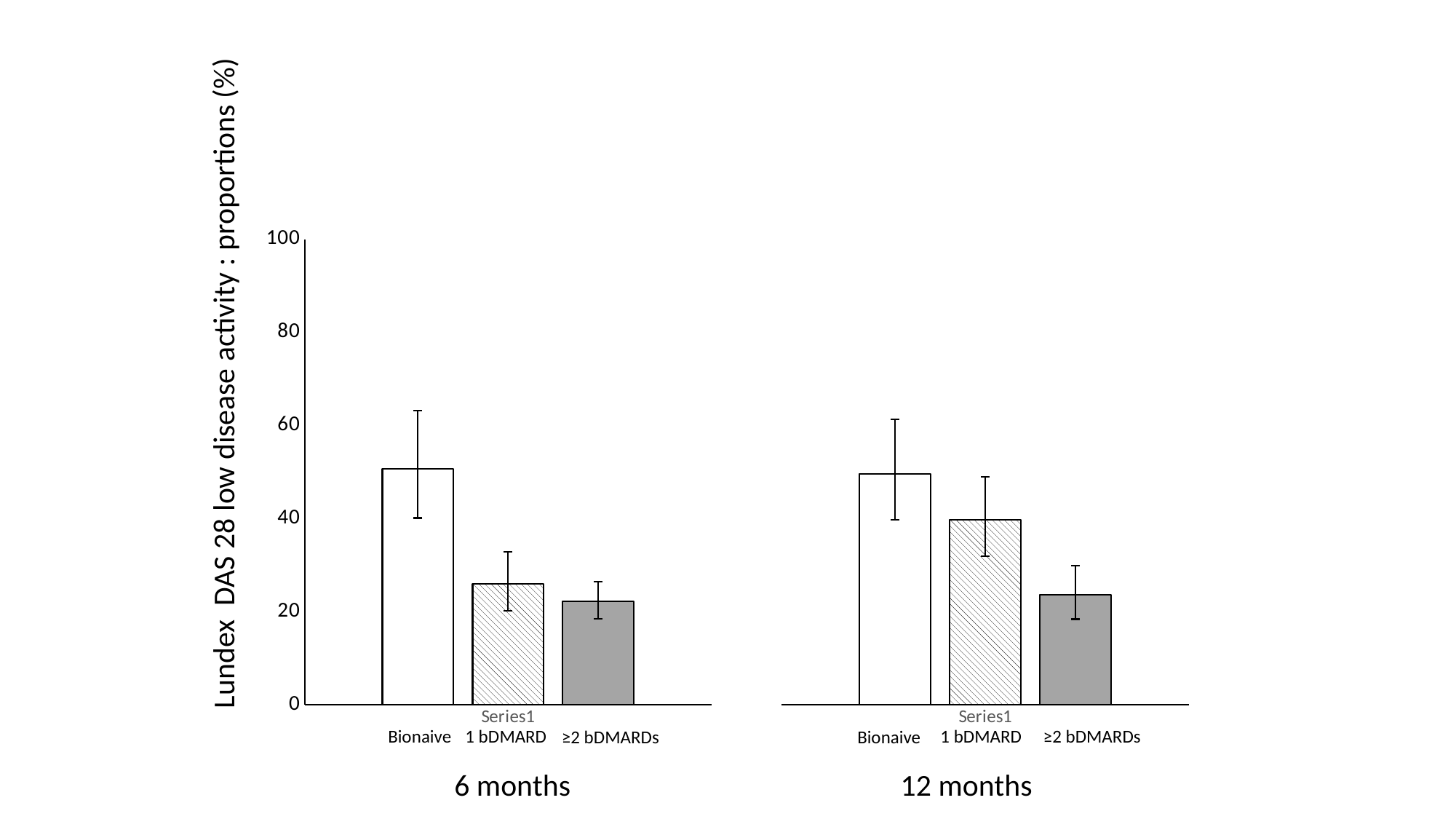

Lundex DAS 28 low disease activity : proportions (%)
### Chart
| Category | Serie 1 | Serie 2 | Serie 3 |
|---|---|---|---|
| | 50.6 | 26.0 | 22.2 |
### Chart
| Category | Serie 1 | Serie 2 | Serie 3 |
|---|---|---|---|
| | 49.4 | 39.6 | 23.5 |Bionaive
1 bDMARD
≥2 bDMARDs
1 bDMARD
≥2 bDMARDs
Bionaive
6 months
12 months
